# Does surgeon volume matter in the outcome of endoscopic inguinal hernia repair?

**DOI:** 10.1007/s00464-016-5001-z

**Published:** 2016-06-22

**Authors:** F. Köckerling, R. Bittner, B. Kraft, M. Hukauf, A. Kuthe, C. Schug-Pass

**Affiliations:** 1 Vivantes Hospital, Berlin, Germany; 2 Winghofer Medicum Hernia Center, Winghofer Straße 42, 72108 Rottenburg am Neckar, Germany; 3 Diakonie Hospital, Department of General and Visceral Surgery, Rosenbergstrasse 38, 70176 Stuttgart, Germany; 4 StatConsult GmbH, Halberstädter Straße 40 a, 39112 Magdeburg, Germany; 5 German Red Cross Hospital, Department of General and Visceral Surgery, Lützerodestraße 1, 30161 Hannover, Germany

**Keywords:** Inguinal hernia, TEP, TAPP, Surgeon volume, Outcome

## Abstract

**Introduction:**

For open and endoscopic inguinal hernia surgery, it has been demonstrated that low-volume surgeons with fewer than 25 and 30 procedures, respectively, per year are associated with significantly more recurrences than high-volume surgeons with 25 and 30 or more procedures, respectively, per year. This paper now explores the relationship between the caseload and the outcome based on the data from the Herniamed Registry.

**Patients and methods:**

The prospective data of patients in the Herniamed Registry were analyzed using the inclusion criteria minimum age of 16 years, male patient, primary unilateral inguinal hernia, TEP or TAPP techniques and availability of data on 1-year follow-up. In total, 16,290 patients were enrolled between September 1, 2009, and February 1, 2014. Of the participating surgeons, 466 (87.6 %) had carried out fewer than 25 endoscopic/laparoscopic operations (low-volume surgeons) and 66 (12.4 %) surgeons 25 or more operations (high-volume surgeons) per year.

**Results:**

Univariable (1.03 vs. 0.73 %; *p* = 0.047) and multivariable analysis [OR 1.494 (1.065–2.115); *p* = 0.023] revealed that low-volume surgeons had a significantly higher recurrence rate compared with the high-volume surgeons, although that difference was small. Multivariable analysis also showed that pain on exertion was negatively affected by a lower caseload <25 [OR 1.191 (1.062–1.337); *p* = 0.003]. While here, too, the difference was small, the fact that in that group there was a greater proportion of patients with small hernia defect sizes may have also played a role since the risk in that group was higher. In this analysis, no evidence was found that pain at rest [OR 1.052 (0.903–1.226); *p* = 0.516] or chronic pain requiring treatment [OR 1.108 (0.903–1.361); *p* = 0.326] were influenced by the surgeon volume.

**Summary:**

As confirmed by previously published studies, the data in the Herniamed Registry also demonstrated that the endoscopic/laparoscopic inguinal hernia surgery caseload impacted the outcome. However, given the overall high-quality level the differences between a “low-volume” surgeon and a “high-volume” surgeon were small. That was due to the use of a standardized technique, structured training as well as continuous supervision of trainees and surgeons with low annual caseload.

In the Guidelines of the European Hernia Society (EHS), the open Lichtenstein and Plug techniques as well as the endoscopic techniques (TEP, TAPP) are recommended as the best evidence-based options for the repair of a primary unilateral inguinal hernia, providing the surgeon is sufficiently experienced in the specific procedure [1, 2]. The Consensus Development Conference of the European Association of Endoscopic Surgery (EAES) and the Guidelines of the International Endohernia Society (IEHS) formulated as a statement that endoscopic groin hernia repair was considered to be more complex than open groin hernia repair [3–5]. Therefore, the learning curve for performing endoscopic inguinal hernia repair is longer than for open Lichtenstein repair and ranges between 50 and 100 procedures, with the first 30–50 being the most critical [1]. The Danish Hernia Database demonstrated on the basis of 14,532 endoscopic/laparoscopic inguinal hernia operations that, in institutions with fewer than 50 endoscopic/laparoscopic inguinal hernia repairs per year, the recurrence rate at 9.97 versus 6.06 % was significantly higher compared with in institutions with more than 50 endoscopic/laparoscopic inguinal hernia operations per year (*p* < 0.0001) [6].

In the Swedish Hernia Registry, there was a significantly higher rate of recurrences for surgeons who carried out one-to-five repairs a year compared with surgeons who performed more repairs [7].

Data on open inguinal hernia surgery in the Statewide Planning and Research Cooperative System Database on 151,322 patients with primary inguinal hernia repairs revealed that low-volume surgeons with fewer than 25 procedures per year had significantly more recurrences than high-volume surgeons with 25 or more procedures per year (hazard ratio 1.23; 95 % confidence interval 1.11–1.36; *p* < 0.001) [8]. Likewise, a retrospective analysis from the Mayo Clinic of 1601 patients with 2410 inguinal hernia repairs in the TEP technique demonstrated that higher annual surgeon volume (>30 vs. 15–30 vs. <15 repairs per year) was associated with improved outcomes as shown by the respective rates for intra- (1 vs. 2.6 vs. 5.6 %) and postoperative (13 vs. 27 vs. 36 %) complications and hernia recurrence (1 vs. 4 vs. 4.3 %) (all *p* < 0.05) [9]. Based on data from the Herniamed Registry [10], this paper now explores whether in a hernia registry too, with several surgeons participating in endoscopic/laparoscopic inguinal hernia surgery, a difference was also identified between those surgeons with fewer than 25 procedures per year compared with surgeons with 25 and more procedures.

## Materials and methods

The Herniamed quality assurance study is a multicenter, internet-based hernia registry [10] into which 460 participating hospitals and surgeons engaged in private practice (Herniamed Study Group) in Germany, Austria, and Switzerland (Status: March 19, 2015) had entered data prospectively on their patients who had undergone hernia surgery. All postoperative complications occurring up to 30 days after surgery are recorded. On one-year follow-up, postoperative complications are once again reviewed when the general practitioner and patients complete a questionnaire. On one-year follow-up, the general practitioner and patients are also asked about any recurrences, pain at rest, pain on exertion, and chronic pain requiring treatment.

In the present analysis, prospective data on male primary unilateral inguinal hernias, operated on in either the total extraperitoneal patch plasty (TEP) or transabdominal patch plasty (TAPP) technique, were analyzed to identify whether surgery had been performed by a surgeon with fewer than 25 or with 25 or more endoscopic/laparoscopic inguinal hernia operations per year. The registry does not, of course, provide any information on the actual experience of individual surgeons.

**Fig. 1:**
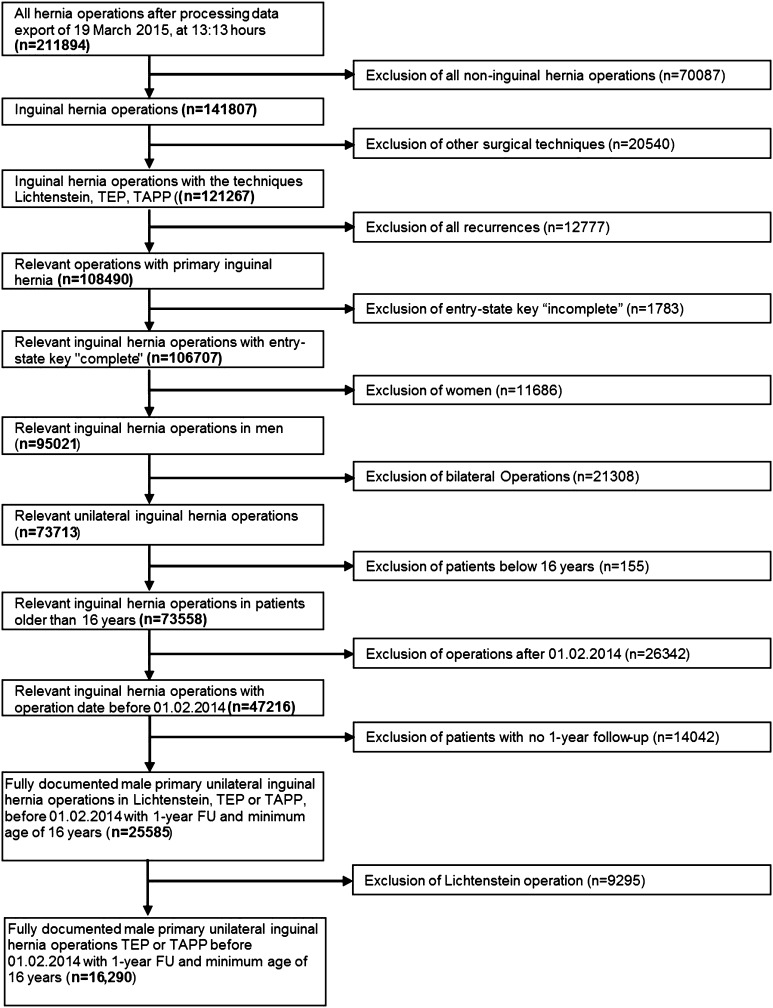
Flowchart of patient inclusion

Inclusion criteria were minimum age of 16 years, male patient, primary unilateral inguinal hernia, TEP or TAPP techniques, and availability of data on one-year follow-up (Fig. 1). In total, 16,290 patients were enrolled between September 1, 2009, and February 1, 2014. Of the participating surgeons, 466 (87.6 %) surgeons had carried out fewer than 25 endoscopic/laparoscopic operations (low-volume surgeons) and 66 (12.4 %) surgeons with 25 or more operations (high-volume surgeons) per year (Table 1). The low-volume surgeons’ group had carried out 9482 (58.2 %), and the high-volume surgeons’ group 6808 (41.8 %) of the total number of endoscopic/laparoscopic procedures (Table 2). The surgeons with fewer than 25 procedures had performed on average 9.47 ± 5.99 operations, and the surgeons with 25 or more procedures 44.12 ± 21.41 operations.

**Table 1:** Number of high- and low-volume surgeons

	Operations per surgeon and year				Total	
	<25		≥25			
	*N*	%	*N*	%	*N*	%
Number of surgeons	466	87.59	66	12.41	532	100.00

**Table 2:** Total number of endoscopic/laparoscopic inguinal hernia repairs and caseload per surgeon

	Operations per surgeon and year				Total	
	<25		≥25			
	*N*	%	*N*	%	*N*	%
Number of endoscopic/laparoscopic operations depending on caseload	9482	58.21	6808	41.79	16,290	100.00

The demographic and surgery-related parameters included age (years), BMI (kg/m^2^), ASA score (I–IV), proportion of medial, lateral, femoral, and scrotal EHS classification as well as the hernia defect size based on EHS classification (Grade I = <1.5 cm, Grade II = 1.5–3 cm, Grade III = >3 cm) [11]. Where an operation entailed several hernia classifications, the latter were summarized as having a “combined” status.

The risk factors included COPD, diabetes, cortisone, immunosuppression, nicotine abuse, coagulopathy or antithrombotic therapy based on antiplatelet or anticoagulant medication. Risk factors were dichotomized, i.e., “yes” if at least one risk factor was positive and “no” otherwise. The dependent variables were intra- and postoperative complication rates, reoperation rates due to postoperative complications, recurrence rates, and rates of pain at rest, pain on exertion, and chronic pain requiring treatment.

All analyses were performed with the software SAS 9.2 (SAS Institute Inc., Cary, NY, USA) and intentionally calculated to a full level of 5 %, i.e., they were not corrected in respect of multiple tests, and each *p* value ≤0.05 represents a significant result. To discern differences between the groups in unadjusted analyses, Fisher’s exact test was used for categorical outcome variables, and the robust *t* test (Satterthwaite) for continuous variables.

To rule out any confounding of data caused by different patient characteristics, the results of unadjusted analyses were verified via multivariable analyses in which, in addition to the surgeon volume, other influence parameters were simultaneously reviewed.

Since the main focus of this analysis is on comparison of surgeon’s caseloads per year (<25/≥25), most of the descriptive statistical analyses in this paper are shown separately for the two groups. All categorical patient data are therefore presented in contingency tables as absolute and relative frequencies for these categories. For continuous data, the mean values and standard deviations are given.

The binary regression model for dichotomous target variables was used to identify the influence of the various factors in multivariable analysis. In addition to the surgeon’s caseload per year (<25/≥25), other potential influence parameters included: ASA score I, II, III, IV, defect size EHS classification I (<1.5 cm), II (1.5–3 cm), III (>3 cm), age, BMI, risk factors, and EHS classification (lateral, medial, scrotal, femoral). As a result, the odds ratios (OR) and corresponding 95 % confidence intervals based on the Wald test are given for estimates. For influence variables with more than two categories, one of these values was used in each case as a reference category. For the continuous variable age (years), the 10-year odds ratio is given and for BMI (kg/m^2^) a 5-point odds ratio. The results are sorted on the basis of influence and presented in tabular form.

## Results

### Comparison of patient collective

With regard to age, patients operated on by surgeons with ≥25 procedures per year had a significantly higher age and were on average one year older (56.1 ± 15.3 vs. 57.1 ± 15.4 years, *p* < 0.001) (Table 3). As regards the BMI, no difference was identified between the patient collectives of surgeons with <25 and ≥25 endoscopic/laparoscopic procedures per year (Table 3).

**Table 3:** Mean age, BMI, and caseload per surgeon

	Operations per surgeon and year		*p*
	<25 OP/year	≥25 OP/year	
Age (year)			
Mean ± STD	56.1 ± 15.3	57.1 ± 15.4	<.001
BMI (kg/m^2^)			
Mean ± STD	25.8 ± 3.3	25.8 ± 3.4	0.757

For the unadjusted tests aimed at identifying a relationship between the caseloads per surgeon and year (<25/≥25) and the categorical influence variables, significant differences were noted for almost all influence variables. Low-volume surgeons operated more often on patients with a low ASA score (e.g., ASA I: 35.9 vs. 28.4 %) as well as with smaller defect sizes (EHS I = <1.5 cm: 15.4 vs. 10.6 %) (Table 4). On the other hand, high-volume surgeons had patients with higher ASA scores (e.g., ASA III/IV: 16.0 vs. 10.9 %), larger defect sizes (e.g., EHS III = >3 cm: 24.1 vs. 20.1 %) as well as scrotal EHS classification (4.3 vs. 1.9 %) (all *p* values <0.001).

**Table 4:** Demographic, patient-related risk factors, and caseload per surgeon

	<25 OP/year		≥25 OP/year		*p*
	*n*	%	*n*	%	
ASA score					
I	3400	35.86	1935	28.42	<.001
II	5051	53.27	3781	55.54	
III/IV	1031	10.87	1092	16.04	
Defect size					
I (<1.5 cm)	1458	15.38	722	10.61	<.001
II (1.5–3 cm)	6122	64.56	4448	65.33	
III (>3 cm)	1902	20.06	1638	24.06	
EHS classification medial					
Yes	3355	35.38	2475	36.35	0.202
No	6127	64.62	4333	63.65	
EHS classification lateral					
Yes	7034	74.18	5103	74.96	0.264
No	2448	25.82	1705	25.04	
EHS classification femoral					
Yes	165	1.74	97	1.42	0.115
No	9317	98.26	6711	98.58	
EHS classification scrotal					
Yes	181	1.91	292	4.29	<.001
No	9301	98.09	6516	95.71	
Risk factor					
Total					
Yes	2468	26.03	1518	22.30	<.001
No	7014	73.97	5290	77.70	
COPD					
Yes	426	4.49	339	4.98	0.148
No	9056	95.51	6469	95.02	
Diabetes					
Yes	438	4.62	271	3.98	0.049
No	9044	95.38	6537	96.02	
Aortic aneurysm					
Yes	37	0.39	17	0.25	0.124
No	9445	99.61	6791	99.75	
Immunosuppression					
Yes	48	0.51	18	0.26	0.017
No	9434	99.49	6790	99.74	
Corticoids					
Yes	82	0.86	40	0.59	0.043
No	9400	99.14	6768	99.41	
Smoking					
Yes	1116	11.77	513	7.54	<.001
No	8366	88.23	6295	92.46	
Coagulopathy					
Yes	105	1.11	82	1.20	0.566
No	9377	98.89	6726	98.80	
Antiplatelet medication					
Yes	558	5.88	454	6.67	0.041
No	8924	94.12	6354	93.33	
Anticoagulation therapy					
Yes	135	1.42	134	1.97	0.007
No	9347	98.58	6674	98.03	

In terms of the risk factors, global analysis, i.e., occurrence of at least one risk factor, also revealed a significant difference (Table 4). In total, 26.0 % of patients operated on by low-volume surgeons had at least one risk factor, while the proportion of those with at least one risk factor operated on by high-volume surgeons was only 22.3 % (*p* = 0.001). That effect was mainly attributable to the difference in the nicotine abuse rate (11.8 vs. 7.5 %; *p* < 0.001). The proportion of patients with antithrombotic therapy based on antiplatelet and anticoagulant treatment was significantly higher in the patient collectively operated on by the high-volume surgeons (Table 4).

### Unadjusted analysis of outcomes by volume

Unadjusted analysis of the relationship between the caseload per surgeon and year did not show any significant difference in the overall intraoperative complication rate between <25 and ≥25 (*p* = 0.526, Table 5). However, surgeons with <25 endoscopic/laparoscopic procedures per year caused significantly more organ injuries, especially vascular injuries (*p* = 0.010, Table 5). As regards the overall postoperative complication rates, low-volume surgeons had, at 2.23 %, a significantly lower rate (*p* < 0.001) compared with the high-volume surgeons at 4.95 % (Table 5). That difference was mainly due to the significantly lower seroma rate in favor of the low-volume surgeons (0.91 vs. 4.20 %; *p* < 0.001). That may be due to the high proportion of inguinal hernias with EHS III (>3 cm) defect size and scrotal classification which was investigated in the subsequent multivariable analysis. No significant difference was found in the rate of postoperative complications, leading to reoperation, which was 0.94 % for the low-volume surgeons and 0.72 % for the high-volume surgeons (*p* = 0.133).

**Table 5:** Unadjusted perioperative and 1-year follow-up outcomes and caseload per surgeon

	<25 OP/year		≥25 OP/year		*p*
	*n*	%	*n*	%	
Intraoperative complications					
Total					
Yes	122	1.29	80	1.18	0.526
No	9360	98.71	6728	98.82	
Bleeding					
Yes	81	0.85	61	0.90	0.777
No	9401	99.15	6747	99.10	
Injuries					
Total					
Yes	72	0.76	29	0.43	0.008
No	9410	99.24	6779	99.57	
Vascular					
Yes	36	0.38	11	0.16	0.010
No	9446	99.62	6797	99.84	
Bowel					
Yes	11	0.12	4	0.06	0.235
No	9471	99.88	6804	99.94	
Bladder					
Yes	7	0.07	8	0.12	0.365
No	9475	99.93	6800	99.88	
Postoperative complications					
Total					
Yes	211	2.23	337	4.95	<.001
No	9271	97.77	6471	95.05	
Bleeding					
Yes	109	1.15	49	0.72	0.006
No	9373	98.85	6759	99.28	
Seroma					
Yes	86	0.91	286	4.20	<.001
No	9396	99.09	6522	95.80	
Infection					
Yes	11	0.12	2	0.03	0.053
No	9471	99.88	6806	99.97	
Bowel injury/anastomotic leakage					
Yes	1	0.01	3	0.04	0.178
No	9481	99.99	6805	99.96	
Impaired wound healing					
Yes	20	0.21	2	0.03	0.002
No	9462	99.79	6806	99.97	
Ileus					
Yes	2	0.02	2	0.03	0.739
No	9480	99.98	6806	99.97	
Reoperation					
Yes	89	0.94	49	0.72	0.133
No	9393	99.06	6759	99.28	
Recurrence on follow-up					
Yes	98	1.03	50	0.73	0.047
No	9384	98.97	6758	99.27	
Pain at rest on follow-up					
Yes	446	4.70	296	4.35	0.283
No	9036	95.30	6512	95.65	
Pain on exertion on follow-up					
Yes	887	9.35	525	7.71	<.001
No	8595	90.65	6283	92.29	
Pain requiring treatment					
Yes	253	2.67	157	2.31	0.146
No	9229	97.33	6651	97.69	

Significant advantages were identified in the recurrence (0.73 vs. 1.03 %; *p* = 0.047) and in the pain on exertion (7.71 vs. 9.35 %; *p* < 0.001) rates in favor of the patient collective operated on by the high-volume surgeons on one-year follow-up (Table 5).

## Multivariable analyses of outcome by volume

### Intraoperative complications

The results obtained with the model used to investigate the effects of the variables related to patient and operation characteristics (caseload per year and surgeon, age, BMI, ASA score, defect size, hernia location as well as the presence of risk factors) on the occurrence of intraoperative complications are illustrated in Table 6 (model matching: *p* = 0.001). The risk of intraoperative complications was affected by scrotal (*p* = 0.011) and medial (*p* = 0.020) EHS classification. Scrotal EHS classification increased the risk of intraoperative complications [OR 2.212 (1.201; 4.073)]. By contrast, medial EHS classification reduced that complication risk [OR 0.577 (0.363; 0.916)].

**Table 6:** Multivariable analysis of intraoperative complications

Parameter	*p* value	Category	*p* value paired	OR estimate	95 % CI	
EHS classification scrotal	0.011	Yes versus no		2.212	1.201	4.073
EHS classification medial	0.020	Yes versus no		0.577	0.363	0.916
ASA score	0.178	I versus II	0.074	0.715	0.495	1.033
		I versus III/IV	0.129	0.660	0.386	1.129
		II versus III/IV	0.708	0.923	0.607	1.403
Caseload per surgeon and year	0.275	<25 versus ≥25		1.174	0.880	1.568
Age (10-year OR)	0.427			1.045	0.937	1.165
EHS classification femoral	0.555	Yes versus no		0.654	0.160	2.678
BMI (5-point OR)	0.719			1.038	0.848	1.270
Defect size	0.808	I versus II	0.903	0.972	0.618	1.530
		I versus III	0.611	0.874	0.520	1.469
		II versus III	0.541	0.899	0.638	1.265
Risk factors	0.878	Yes versus no		0.974	0.697	1.361
EHS classification lateral	0.948	Yes versus no		1.017	0.611	1.691

However, no evidence was found that an individual surgeon’s caseload (<25 vs. ≥25 endoscopic/laparoscopic inguinal hernia repairs per year) influenced the intraoperative complication rate [OR 1.174 (0.880–1.568); *p* = 0.275].

### Postoperative complications

The results obtained with the model used to investigate the postoperative complication rate are presented in Table 7 (model matching: *p* < 0.001). The risk of postoperative complications was negatively impacted by high-volume surgeons, scrotal hernias, higher age, and larger defects. That risk declined when a surgeon had performed fewer than 25 procedures per year [OR 0.463 (0.388; 0.554); *p* < 0.001]. Scrotal EHS classification increased the risk of occurrence of a postoperative complication [OR 2.076 (1.444; 2.984); *p* < 0.001]. Equally, a higher age [10-year OR 1.114 (1.041; 1.192); *p* = 0.002] increased the postoperative complication rate. Finally, the presence of a smaller defect size reduced the postoperative complication rate [I vs. II: OR 0.700 (0.505; 0.970); *p* = 0.032. I vs. III: OR 0.580 (0.406; 0.830); *p* = 0.003].

**Table 7:** Multivariable analysis of postoperative complications

Parameter	*p* value	Category	*p* value paired comparison	OR estimate	95 % CI	
Caseload per surgeon and year	<.001	<25 versus ≥ 25		0.463	0.388	0.554
EHS classification lateral	<.001	Yes versus no		0.471	0.350	0.633
BMI (5-point OR)	<.001			0.746	0.649	0.858
EHS classification scrotal	<.001	Yes versus no		2.076	1.444	2.984
EHS classification medial	<.001	Yes versus no		0.566	0.423	0.758
Age (10-year OR)	0.002			1.114	1.041	1.192
Defect size	0.010	I versus II	0.032	0.700	0.505	0.970
		I versus III	0.003	0.580	0.406	0.830
		II versus III	0.072	0.829	0.676	1.017
ASA score	0.092	I versus II	0.764	1.035	0.827	1.295
		I versus III/IV	0.135	0.786	0.573	1.078
		II versus III/IV	0.029	0.759	0.593	0.972
Risk factors	0.761	Yes versus no		1.033	0.838	1.273
EHS classification femoral	0.990	Yes versus no		0.996	0.516	1.921

Likewise, medial and lateral EHS classification and higher BMI reduced the risk of postoperative complications. Lateral [OR 0.471 (0.350; 0.633); *p* < 0.001] or medial EHS classification [OR 0.566 (0.423; 0.758); *p* < 0.001] as well as a five-point higher BMI [five-point OR 0.746 (0.649; 0.858); *p* < 0.001] reduced the postoperative complication rate.

### Recurrence

Table 8 presents the results of multivariable analysis of factors impacting recurrence on one-year follow-up (model matching: *p* = 0.001). BMI proved to be the strongest influence factor (*p* = 0.004). A five-point higher BMI increased the recurrence rate [five-point OR 1.342 (1.098; 1.640)]. Likewise, medial EHS classification significantly increased the recurrence rate [OR 1.690 (1.077; 2.652); *p* = 0.022]. The surgical volume of the individual surgeons also had a significant influence on the risk (*p* = 0.023). Surgeons with <25 endoscopic/laparoscopic operations per year had a higher recurrence rate [OR 1.494 (1.056; 2.115); *p* = 0.023]. With a prevalence of 0.9 %, this would correspond to 11 recurrences for 1000 operations by surgeons with <25 endoscopic/laparoscopic inguinal hernia repairs per year compared to seven recurrences for ≥25 operations per year.

**Table 8:** Multivariable analysis of recurrence

Parameter	*p* value	Category	*p* value paired comparison	OR estimate	95 % CI	
BMI (5-point OR)	0.004			1.342	1.098	1.640
EHS classification medial	0.022	Yes versus no		1.690	1.077	2.652
Caseload per surgeon and year	0.023	<25 versus ≥25		1.494	1.056	2.115
ASA score	0.090	I versus II	0.195	0.758	0.498	1.152
		I versus III/IV	0.028	0.510	0.279	0.931
		II versus III/IV	0.103	0.673	0.418	1.083
EHS classification scrotal	0.173	Yes versus no		1.779	0.777	4.073
Age (10-year OR)	0.342			0.940	0.828	1.068
Defect size	0.532	I versus II	0.315	1.273	0.795	2.039
		I versus III	0.724	1.105	0.636	1.921
		II versus III	0.488	0.868	0.581	1.296
EHS classification femoral	0.735	Yes versus no		1.221	0.383	3.894
EHS classification lateral	0.777	Yes versus no		0.935	0.586	1.491
Risk factors	0.996	Yes versus no		1.001	0.680	1.474

### Pain at rest

Analysis of the results obtained on investigating pain at rest on one-year follow-up is illustrated in Table 9 (model matching: *p* < 0.001). The defect size proved to be the strongest influence factor here (*p* < 0.001). A small defect size increased the risk of pain at rest on follow-up [I vs. II: OR 1.671 (1.382; 2.022); I vs. III: OR 2.205 (1.702; 2.857); II vs. III: OR 1.319 (1.065; 1.634); *p* = 0.011]. Equally, BMI and age had a highly significant impact on pain at rest (in each case *p* < 0.001). A five-point higher BMI increased pain at rest [five-point OR 1.230 (1.114; 1.359)]. Conversely, higher age [10-year OR 0.890 (0.841; 0.941)] reduced the risk of pain at rest. Finally, femoral EHS classification increased the risk of pain at rest [OR 1.772 (1.106; 2.839); *p* = 0.017]. The number of surgical procedures performed by a surgeon per year did not impact the risk of onset of pain at rest.

**Table 9:** Multivariable analysis of pain at rest

Parameter	*p* value	Category	*p* value paired	OR estimate	95 % CI	
Defect size	<.001	I versus II	<.001	1.671	1.382	2.022
		I versus III	<.001	2.205	1.702	2.857
		II versus III	0.011	1.319	1.065	1.634
BMI (5-point OR)	<.001			1.230	1.114	1.359
Age (10-year OR)	<.001			0.890	0.841	0.941
EHS classification femoral	0.017	Yes versus no		1.772	1.106	2.839
ASA score	0.072	I versus II	0.035	0.822	0.685	0.986
		I versus III/IV	0.056	0.751	0.559	1.008
		II versus III/IV	0.473	0.913	0.713	1.170
EHS classification lateral	0.231	Yes versus no		1.164	0.908	1.491
Risk factors	0.267	Yes versus no		1.107	0.925	1.323
Caseload per surgeon and year	0.516	<25 versus ≥25		1.052	0.903	1.226
EHS classification medial	0.785	Yes versus no		1.031	0.827	1.286
EHS classification scrotal	0.868	Yes versus no		1.043	0.632	1.722

### Pain on exertion

Analysis of the results obtained on investigating pain on exertion on one-year follow-up is summarized in Table 10 (model matching: *p* < 0.001). Pain on exertion was significantly and negatively influenced by the defect size, BMI, and caseload of <25 procedures per surgeon and year. The risk of pain on exertion increased for smaller defect sizes [I vs. II: OR 1.358 (1.173; 1.572); *p* < 0.001; I vs. III: OR 1.673 (1.376; 2.035); *p* < 0.001; II vs. III: OR 1.232 (1.053; 1.443); *p* = 0.009] and for a five-point higher BMI [five-point OR 1.179 (1.092; 1.272); *p* < 0.001]. Likewise, a caseload <25 procedures per year significantly increased the risk of onset of pain on exertion [OR 1.191 (1.062; 1.337); *p* = 0.003]. A higher age [10-year OR 0.772 (0.741; 0.804); *p* < 0.001] reduced onset of pain on exertion.

**Table 10:** Multivariable analysis of pain on exertion

Parameter	*p* value	Category	*p* value paired comparison	OR estimate	95 % CI	
Age (10-year OR)	<.001			0.772	0.741	0.804
Defect size	<.001	I versus II	<.001	1.358	1.173	1.572
		I versus III	<.001	1.673	1.376	2.035
		II versus III	0.009	1.232	1.053	1.443
BMI (5-point OR)	<.001			1.179	1.092	1.272
Caseload per surgeon and year	0.003	<25 versus ≥25		1.191	1.062	1.337
ASA score	0.086	I versus II	0.036	0.869	0.761	0.991
		I versus III/IV	0.097	0.823	0.655	1.036
		II versus III/IV	0.600	0.948	0.777	1.157
Risk factors	0.168	Yes versus np		1.100	0.961	1.259
EHS classification medial	0.547	Yes versus no		1.053	0.890	1.247
EHS classification femoral	0.719	Yes versus no		1.082	0.703	1.666
EHS classification lateral	0.854	Yes versus no		1.018	0.845	1.226
EHS classification scrotal	0.951	Yes versus no		1.012	0.696	1.472

### Chronic pain requiring treatment

The results obtained on investigating chronic pain requiring treatment are presented in Table 11 (model matching: *p* < 0.001). The hernia defect size proved to be the strongest influence factor here (*p* < 0.001). A smaller defect size increased the risk of onset of chronic pain requiring treatment on follow-up [I vs. II: OR 2.084 (1.642; 2.644); I vs. III: OR 2.567 (1.832; 3.597)]. Equally, age and BMI had a highly significant effect on chronic pain requiring treatment (*p* < 0.001). Higher age [10-year OR 0.810 (0.752; 0.872)] reduced onset of chronic pain requiring treatment. A five-point higher BMI increased the risk of pain [five-point OR 1.339 (1.183; 1.516)].

**Table 11:** Multivariable analysis of pain requiring treatment

Parameter	*p* value	Category	*p* value paired	OR estimate	95 % CI	
Defect size	<.001	I versus II	<.001	2.084	1.642	2.644
		I versus III	<.001	2.567	1.832	3.597
		II versus III	0.162	1.232	0.919	1.651
Age (10-year OR)	<.001			0.810	0.752	0.872
BMI (5-point OR)	<.001			1.339	1.183	1.516
ASA score	0.095	I versus II	0.199	0.855	0.673	1.086
		I versus III/IV	0.030	0.652	0.442	0.960
		II versus III/IV	0.105	0.762	0.549	1.059
Risk factors	0.144	Yes versus no		1.192	0.942	1.509
Operation (OR/year)	0.326	<25 versus ≥25		1.108	0.903	1.361
EHS classification femoral	0.352	Yes versus no		1.386	0.698	2.752
EHS classification lateral	0.389	Yes versus no		1.159	0.828	1.622
EHS classification scrotal	0.633	Yes versus no		1.170	0.615	2.225
EHS classification medial	0.964	Yes versus no		1.007	0.746	1.359

## Discussion

The learning curve associated with endoscopic/laparoscopic inguinal hernia surgery requiring 50–100 procedures is longer than that involving the open Lichtenstein operation [1]. Under the supervision of experienced laparoscopic surgeons, young trainees can master the learning curve with good results [12]. Apart from the learning curve, other aspects increasingly discussed in surgery are the impact of the caseload of the treating institution and of the individual surgeon. In the hernia surgery setting, this topic has been addressed so far in three studies on, in each case, open incisional hernia surgery [13], open inguinal hernia surgery [8], and endoscopic inguinal hernia surgery in TEP technique [9]. All three studies identified a significant relationship between the individual surgeon’s caseload per year and patient outcome.

In the present paper, the results obtained for perioperative complications and 1-year follow-up of endoscopic/laparoscopic inguinal hernia surgery based on data from the Herniamed Registry were analyzed to ascertain whether the number of operations per surgeon and year (<25 vs. ≥25) impacted the outcome. Differences were identified first of all on comparing the patient collectives undergoing surgery. The high-volume surgeons (≥25 operations per year) operated on significantly more patients with higher ASA score, larger defect size, and scrotal hernia. Likewise, patients operated on by the high-volume surgeons had received significantly more often effective treatment with platelet aggregation inhibitors and coumarin derivatives.

Overall, patients operated on by high-volume surgeons had thus a significantly higher risk profile with, accordingly, significantly more postoperative complications observed in the patients operated on by high-volume surgeons. That this, nonetheless, did not result in more postoperative complications requiring reoperation, but rather in a higher rate of seromas amenable to conservative treatment, attesting to the skill of experienced surgeons in mastering their patients’ higher-risk profile. The greater proportion of seromas in the patient group treated by the high-volume surgeons can also be explained by the significantly larger proportion of Grade III hernias (defect size >3 cm) and scrotal hernias. Apart from that, in patients operated on by low-volume surgeons (<25 operations per year), there were significantly more cases of secondary bleeding and impaired wound healing, but at 1.15 versus 0.72 and 0.21 versus 0.03 %, respectively, that difference was very small.

Univariable analysis of the findings on 1-year follow-up revealed that patients operated on by the low-volume surgeons had a significantly higher recurrence rate and pain on exertion rate but here, too, the differences at 1.03 versus 0.73 and 9.35 versus 7.71 %, respectively, were small. Univariable analysis of data for pain at rest and chronic pain requiring treatment did not reveal any differences.

Multivariable analysis revealed that scrotal hernia and large defect size had a significant influence on onset of a postoperative complication. The risk of occurrence of a postoperative complication was less in association with medial or lateral EHS classification, higher BMI value and, interestingly, for surgeons with a caseload of fewer than 25 operations per year. The only explanation that can be given for the latter finding is that surgeons with fewer than 25 procedures per year generally had operated on patients with a lower-risk profile.

Multivariable analysis of the influence variables impacting recurrence showed that higher BMI, medial EHS classification, and a caseload of fewer than 25 procedures per year were associated with a higher risk.

Pain at rest was revealed by multivariable analysis to be negatively affected by a smaller defect size, higher BMI value, and femoral EHS classification. Older patients were found to have a lower risk of onset of pain at rest.

Likewise, multivariable analysis showed that onset of pain on exertion was negatively influenced by smaller defect size, higher BMI value, and additionally by a caseload of fewer than 25 surgical procedures per year. Higher age was also found to be associated with a lower risk of pain on exertion.

Equally, chronic pain requiring treatment was negatively impacted by a smaller hernia defect and higher BMI, with here, too, a lower risk in older patients. The caseload per year did not affect that outcome criterion.

As such, the registry data presented in this paper for endoscopic/laparoscopic inguinal hernia surgery confirm that the annual caseload of the individual surgeons exerted a certain amount of influence on the outcome but the differences were not as pronounced as in the publication by the Mayo Clinic [9]. This is no doubt due to the fact that in the German system even trained surgeons who have less experience of a surgical technique work under the supervision of an experienced surgeon, thus assuring that in such settings, too, good results can be achieved [12]. Based on the experience of the surgeon, also of the trained surgeon, the Chairman of a Department of Surgery decides whether the surgeon can perform the operation alone or under the guidance of a more experienced colleague. The registry does not, of course, provide any information on the actual experience of individual surgeons. It must also be borne in mind that unlike the National Danish and Swedish Registries the data in the Herniamed Registry are collected only from hospitals with a special interest in hernia surgery. Furthermore, the high-volume surgeons were responsible for the more difficult cases, i.e., more advanced hernias. The difference would have probably been much greater if the study had been randomized.

In summary, it can be stated that with regard to the quality parameters recurrence rate and pain on exertion, a “low-volume surgeon” achieves slightly worse results than a “high-volume” surgeon, but overall can assure a high-quality level in endoscopic/laparoendoscopic inguinal hernia surgery. The preconditions for a good outcome, also in routine clinical settings and, in particular, for trainee surgeons or surgeons with lower annual caseloads, are the use of a standardized technique, a structured training program, and close supervision of trainees and of surgeons with lower caseloads.
